# Sephadex^®^ LH-20, Isolation, and Purification of Flavonoids from Plant Species: A Comprehensive Review

**DOI:** 10.3390/molecules25184146

**Published:** 2020-09-10

**Authors:** Javad Mottaghipisheh, Marcello Iriti

**Affiliations:** 1 Department of Pharmacognosy, Faculty of Pharmacy, University of Szeged, Eötvös u. 6, 6720 Szeged, Hungary; 2 Department of Agricultural and Environmental Sciences, Milan State University, via G. Celoria 2, 20133 Milan, Italy

**Keywords:** flavonoids, size-exclusion chromatography, Sephadex^®^ LH-20, isolation, purification

## Abstract

Flavonoids are considered one of the most diverse phenolic compounds possessing several valuable health benefits. The present study aimed at gathering all correlated reports, in which Sephadex^®^ LH-20 (SLH) has been utilized as the final step to isolate or purify of flavonoid derivatives among all plant families. Overall, 189 flavonoids have been documented, while the majority were identified from the Asteraceae, Moraceae, and Poaceae families. Application of SLH has led to isolate 79 flavonols, 63 flavones, and 18 flavanones. Homoisoflavanoids, and proanthocyanidins have only been isolated from the Asparagaceae and Lauraceae families, respectively, while the Asteraceae was the richest in flavones possessing 22 derivatives. Six flavones, four flavonols, three homoisoflavonoids, one flavanone, a flavanol, and an isoflavanol have been isolated as the new secondary metabolites. This technique has been able to isolate quercetin from 19 plant species, along with its 31 derivatives. Pure methanol and in combination with water, chloroform, and dichloromethane have generally been used as eluents. This comprehensive review provides significant information regarding to remarkably use of SLH in isolation and purification of flavonoids from all the plant families; thus, it might be considered an appreciable guideline for further phytochemical investigation of these compounds.

## 1. Introduction

Flavonoids are considered as one of the most varied phenolic compounds. Different derivatives of these secondary metabolites, which are naturally synthesized in higher plants and microbial sources, possess extensive beneficial properties for human health. Many studies have assessed pharmacological and bioactivities of these compounds. Along with those effects, the importance of these compounds is mainly related to their ability in the scavenging of free radicals, hence possessing high antioxidant activity [1,2].

Isolation of flavonoids have been majorly carried out by hiring chromatographic methods. These techniques have been developed by the Noble laureates in chemistry at 1952 Archer John Porter Martin and Richard Laurence Millington Synge for their invention of partition chromatography [3]. These methods are the most remarkable separation techniques, which are extensively applied in natural product chemistry analysis for both analytical and preparative purposes. The chromatographic methods functionally separate the mixtures according to physical properties of their constituents.

Silica gel chromatography has been widely used in the isolation and characterization of these compounds. They can be separated according to the polarity, while normal phase and reversed phase (C18 silica gel) are applied for isolation of low to moderate polar and high polar flavonoids (e.g., glycosylated forms), respectively. The ability of polyamide to make hydrogen bonds with hydroxyl groups of flavonoids, depending on the numbers and positions of this groups, are the basis of the isolation process in this technique. The recent advanced techniques, including High-performance liquid chromatography (HPLC), high-speed counter current chromatography (HSCCC), molecular imprinting technology (MIT), droplet counter-current chromatography (DCCC), along with older methods medium pressure liquid chromatography (MPLC), circular liquid chromatography (CLC), and centrifugal preparative thin layer chromatography (CPTLC), are the applied techniques to isolate flavonoids [2,4,5]. However, GC-MS, HPLC-ESI-Q-TOF-MS, HPLC-PdAD-ESI-MS/MS, LC-MS, LC-MS/MS, and ultra-HPLC (UHPLC), have mostly been employed to analytical analysis of flavonoids from plant species [5]. The acidity of the extraction solvents have been reported a direct correlation with flavonoid contents, whereas in a study on *Vitis vinifera*, total flavonoid contents enhanced (from 20.63 to 46.77 mg/g) by addition of HCl (from 0 to 1%) [6]. In another analytical research, the content of spinacetin 3-gentiobioside was the highest in a pH of 2.5, compared to the applied acidity media (7.25 and 12), with the same other parameters in the extraction procedure [7].

Size-exclusion chromatography is considered a kind of partition chromatography, which is able to isolate compounds according to various molecular sizes. Gel-permeation, gel-exclusion, gel filtration, and molecular-sieve chromatography are the alternative definitions of this technique. The diameter and pore size of packed materials, choosing a proper eluent as mobile phase, and length of the used column are the significant parameters for effectively separation of a mixture by size exclusion chromatography [8].

The first application of size-exclusion procedure refers to separation of peptides from amino acids on a column packed with starch [9]. The Pharmacia company (Stockholm, Sweden) developed dextran crosslinked with epichlorohydrin with tradename of “Sephadex^®^” [10]. Initially, Sephadex^®^ comprised irregular particles, then was synthesized as porous spheres [11].

Nuclear magnetic resonance (NMR), mass spectrometry (MS), spectrophotometric ultra-violet (UV), and infrared (IR) techniques providing spectroscopic spectra of flavonoids, have been exploited to elucidate the flavonoid structures. Moreover, their physical characteristics such as melting point (mp), circular dichroism (CD), and optical rotatory power ([α]_D_) can be applied to identify the flavonoids [5].

However, the new techniques have facilitated isolation and identification of flavonoids from specifically plant resources, the classical methods particularly Sephadex^®^ LH-20 (SLH) has been widely utilized, due to being inexpensive, convenient, rapid, and efficient. For the first time, this review comprehensively gathered all the existing information about the application of SLH in isolation or purification of diverse range of flavonoid derivatives, where this method has been used as the last separation step. The keywords of “flavonoid” and “Sephadex” have been applied to search the correlated published data through databases including PubMed and Web of Science (last search: 27 June 2020).

## 2. Isolation of Various Flavonoid Classifications by Sephadex^®^ LH-20

**Figure 1:**
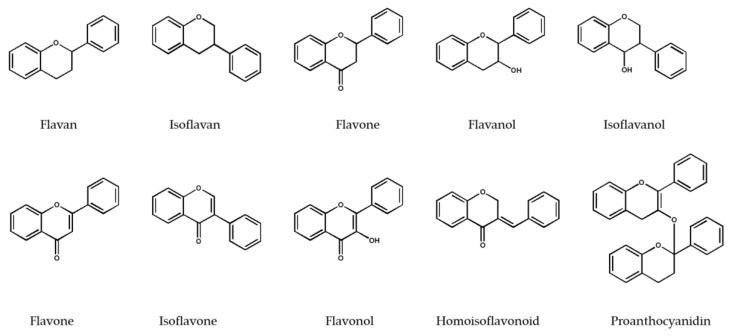
The basic chemical structures of the flavonoid classes isolated or purified by applying Sephadex^®^ LH-20.

Overall, 190 flavonoid derivatives have been isolated or purified by utilization of SLH from 40 various plant families. This method has been able to isolate or purify seven major flavonoid classifications, including flavan and isoflavan, flavanone, flavanol and isoflavanol, flavone and isoflavone, flavonol, homoisoflavonoid, and proanthocyanidin derivatives (Table S1). In general, one flavan and an isoflavan, 18 flavanones, eight flavanols and one isoflavanol, 63 flavones and five isoflavones, 79 flavonols, 10 homoisoflavonoids, and three proanthocyanidins have been isolated and identified. The basic chemical structures of the flavonoid classifications have been illustrated in Figure 1.

**Figure 2:**
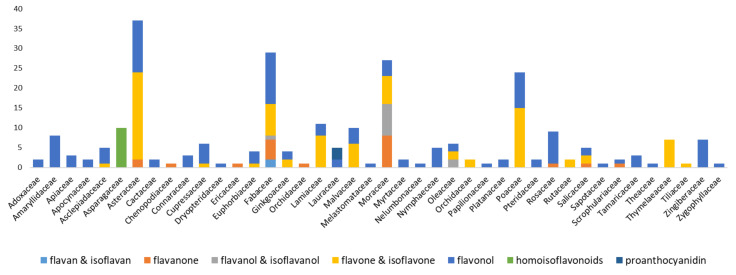
Number of flavonoid derivatives isolated or purified by Sephedex^®^ LH-20.

The most isolated flavonoids have been identified from Asteraceae family including 37 different flavonoids, besides the plants belonging to Moraceae and Poaceae with 27 and 24 possessed more flavonoids, respectively (Figure 2). The species in families Asteraceae with 22 flavones, and Asteraceae and Fabaceae with 13 flavonols were the richest; whilst whole 10 and three isolated homoisoflavonoids and proanthocyanidins have been isolated from Asparagaceae and Lauraceae families, respectively.

Overall, 17 different eluents have been applied to isolate/purify flavonoids through SLH column. Pure methanol and its mixtures specifically in combination with water have been exploited as the most prevalent eluents in isolation of 71 and 67 flavonoids, respectively (Table 1).

### 2.1. Flavan and Isoflavan Derivatives

Among 15 isolated flavonoids from chloroform extract of *Dalbergia cochinchinensis* herb, one flavan namely 6,4′-dihydroxy-7-methoxy-flavan (**1**) and an isoflavan mucronulatol (**2**) have been purified by applying SLH with dichloromethane‒methanol (1:1) as the eluting solvent [12].

### 2.2. Flavanone Derivatives

Dihydrowogonin (**3**) is a 5,7-dihydroxy-8-methoxyflavanone which has been isolated from dichloromethane extract of *Chenopodium procerum* aerial parts. Methanol was applied as solvent system to isolate the mentioned flavanone via SLH [13].

Isolation of naringenin (**4**) has been carried out from three plant species. *n*-Butanol extract obtained from *Paulownia tomentosa* bark by applying methanol‒water (1:1, 1:3) [14], chloroform fraction of *Dalbergia cochinchinensis* herb with dichloromethane‒water (1:1) [12], and ethyl acetate extract gained from wooden part of *Populus davidiana* by using methanol‒water (3:1, 1:1, 1:3) as elution solvents [15]. One glycosylated derivative of naringenin called naringenin 7-*O*-β-glucopyranoside (syn. prunin) (**5**) has been furtherly isolated from hydro-methanolic extract (80%) of leaf and flower of Hawthorn (*Crataegus* spp.) by increasing ratio of methanol (40 to 70%) in water by applying SLH [16].

Jung et al. [17] subjected ethyl acetate extract of root bark of *Morus alba* to isolate major constituents. Flavanones including sanggenol Q (**6**), sanggenol F (**7**) [17], a new compound sanggenon U (**8**), and kuwanon E (**9**) by using methanol‒water (8:2), along with euchrenone a7 (**10**) with methanol‒water (7:3) as SLH eluents have been isolated and purified [18]. In another study, three other flavanones namely sanggenon J (**11**), sanggenon F (**12**), and sanggenol A (**13**) have also been isolated from the root bark ethyl acetate extract of *M. alba*, where the samples were eluted with methanol and methanol‒water (1:1) as eluents through SLH [19].

Dichloromethane‒methanol (1:1) has been used as eluent to isolate pinocembrin (**14**) from ethyl acetate extract of *Corema album* and petroleum ether fraction of *Dalbergia cochinchinensis* herb. This compound is a 5,7-dihydroxyflavanone and has been extracted from honey, propolis, ginger roots, etc. were reported as a potential natural drug to treat ischemic stroke, and for its anti-inflammatory and neuroprotective effects [20,21].

Liquiritigenin (**15**) and alpinetin (**16**) from chloroform, and 7,8-dihydroxyflavanone (**17**) from ethyl acetate extracts of *Dalbergia cochinchinensis* herb have been previously isolated by eluting dichloromethane‒methanol (1:1) through SLH [12]. Methanolic extract obtained from the aerial parts of *Taraxacum mongolicum* have been chromatographed and finally two flavanones hesperidin (**18**) and 4′,5,7-trihydroxy-3′-methoxyflavanone (**19**) were purified by SLH (eluent: methanol) [22]. Another aglycone flavanone (2*S*)-homoeriodictyol (**20**) has been furtherly isolated from methanolic extract of the whole parts of *Dendrobium ellipsophyllum* applying SLH eluting with acetone [23].

### 2.3. Flavanol and Isoflavanol Derivatives

In general, eight flavanol (**21**–**28**) and a novel isoflavanol (**29**) have been isolated and purified by SLH as the final chromatographic step from families including Oleaceae, Moraceae, and Fabaceae. Phytochemical investigation of ethyl acetate extract of *Chionanthus retusus* flowers led to the isolation of aromadendrin (**21**) and taxifolin (syn. dihydroquercetin) (**22**) using SLH (eluent: methanol‒water 8:2) as the last separation step [24]. The aforementioned aglycone flavanols (**21**,**22**), along with two glycosylated taxifolin namely taxifolin 7-glucoside (**23**) and 6-*p*-hydroxybenzyl taxifolin-7-*O*-β-d-glucoside (**24**), and two other aglycones gericudranin E (**27**) and gericudranin C (**28**), have been furtherly isolated from *Cudrania tricuspidata* aqueous extract of bark utilizing methanol‒water (1:1) as eluting solvent [25].

2,3-*trans*-Dihydromorin (**25**) [19] and a novel flavanol (2*R*,3*S*)-guibourtinidol-3-*O*-α-d-apiofuranosyl-(1→6)-*O*-β-d-glucopyranoside (**26**) [26] have been previously isolated from ethyl acetate and *n*-butanol extracts of *Morus alba* root barks via gel filtration SLH column with methanol and methanol‒water (3:2) as eluents, respectively. Awouafack et al. [27] hired SLH to isolate a new isoflavanol namely kotstrigoisoflavanol (**29**) from methanolic extract of *Kotschya strigosa* fruit.

### 2.4. Flavone and Isoflavone Derivatives

The 63 flavone (**30**‒**92**) and five isoflavone (**93**‒**97**) derivatives isolated by using SLH as the last separation stage illustrated that this technique plays an effective role in extraction of these compounds.

The simple flavone (**30**) and its derivative 4′-hydroxy-5-methoxyflavone (**33**) have been isolated from *Imperata cylindrica*, whilst ethyl acetate extracts of rhizome were finally chromatographed via SLH with dichloromethane‒methanol (1:1) as eluent system [28]. Ethyl acetate and ethanolic extracts gained from stem bark and aerial parts of *Albizzia julibrissin* and *Athrixia phylicoides* were extracted to isolate two aglycone flavones of 3′,4′,7-trihydroxyflavone (**31**) [29] and 5-hydroxy-6,7,8,3′,4′,5′-hexamethoxyflavon-3-ol (**32**) [30], respectively, through a separation procedure with SLH (eluent: methanol).

A well-known flavone luteolin (**34**) (3,4,5,7-tetrahydroxy flavone) possessing several health benefits, such as anti-cancer [31], cardio-protective [32], anti-inflammation, and anti-allergy [33] effects, has been isolated and purified from 11 plant species applying SLH as the final step: from hydroethanolic (70%) extract of *Brachychiton acerifolius* leaf (eluent: methanol‒water 1:1) [34], ethyl acetate extracts of *Thymus praecox* aerial part [35], *Ginko biloba* leaf (eluent: methanol) [36], *Rosmarinus officinalis* sprig (eluent: methanol‒water 1:1) [37], *Chamaemelum nobile* flower (eluent: methanol‒dichloromethane 1:1) [38], *Populus davidiana* wood (eluent: methanol‒water 3:1, 1:1, 1:3) [15], and *Solenostemon monostachys* aerial part (eluent: *n*-hexane‒ethyl acetate 3:7, 2:8, 1:9; ethyl acetate; ethyl acetate‒methanol 1:9, 2:8, 4:6, 5:5) [39], aqueous fraction of *Phlomis bruguieri* aerial part (eluent: *n*-hexane‒MeOH‒acetone 30:60:10) [40], methanolic extracts of *Taraxacum mongolicum* aerial part (eluent: methanol) [22] and *Dendrobium ellipsophyllum* whole plant part (eluent: acetone) [23], and *n*-butanol extract of xylem part of *Populus tomentosa* (eluent: methanol‒water 1:1, 1:3) [41].

Moreover, 7-methoxy luteolin (**35**) has been isolated from ethyl acetate extract of *Onopordum alexandrinum* seeds via SLH as the final step with methanol‒water (9:1) as eluting solvent [42]. Overall, five glycosylated luteolin (**36**‒**40**) have been purified applying gel filtration chromatography. Orientin (**36**) which is luteolin 8-*C*-glucoside was finally isolated by SLH (eluent: methanol) from petroleum ether extract of *Indocalamus latifolius* leaf [43].

Cynaroside (**37**) as luteolin 7-*O*-β-d-glucoside have been previously isolated from six plant species: ethyl acetate extracts of *Tridax procumbens* whole part [44] and *Salvia macrosiphon* aerial part (eluent: methanol) [45], hydro-methanolic (80%) portion of *Tilia rubra* leaf (eluent: methanol‒water 8:2), hydroethanolic extracts of leaf of *Olea europaea* (eluent: ethanol 0–50% in water) [46] and *Brachychiton acerifolius* (eluent: methanol‒water 1:1) [34], and chloroform extract obtained from *Citrus unshiu* peel (eluent: methanol‒water 1:1) [47].

From the methanolic extract of *Taraxacum mongolicum* aerial part eluting with methanol through SLH, luteolin-7-*O*-β-d-galactopyranoside (**38**) and luteolin-7-*O*-β-d-glucopyranoside (**39**) [22], and luteolin-4′-*O*-β-glucoside (**40**) from hydroethanolic (50%) extract of *Olea europaea* leaf (eluent: ethanol 0‒50% in water) [46] have been furtherly isolated.

Apigenin (**41**), characterized as 4′,5,7,-trihydroxyflavone is considered as a natural flavone, and rich in several fruits, vegetables and medicinal plants possessing numerous pharmacological potencies, such as anti-inflammatory, antioxidant, antibacterial, antiviral, antidiabetic, antidepressant, and anticancer activities, and the treatment of amnesia and Alzheimer’s disease, and insomnia [48,49,50,51,52]. SLH has been capable to isolate this natural product from hydroethanolic extracts of *Brachychiton acerifolius* leaf (eluent: methanol‒water 1:1) [34] and *Saccharum officinarum* sugarcane top (eluent: chloroform‒methanol 1:1) [53], ethyl acetate fractions of *Chamaemelum nobile* flowers (eluent: methanol‒dichloromethane 1:1) [38], and *Solenostemon monostachys* aerial part (eluent: *n*-hexane‒ethyl acetate 3:7, 2:8, 1:9; ethyl acetate; ethyl acetate‒methanol 1:9, 2:8, 4:6, 5:5) [39], *n*-butanol extract of xylem of *Populus tomentosa* (eluent: methanol‒water 1:1, 1:3) [41], and aqueous extract of *Phlomis bruguieri* aerial part (eluent: *n*-hexane‒methanol‒acetone 30:60:10) [40].

From leaf hydroethanolic (70%) extract of *Brachychiton acerifolius*, apigenin-7-*O*-α-rhamnosyl (1→2)-β-D-glucuronide (**42**), apigenin-7-*O*-β-d-glucoside (**43**), and apigenin-7-*O*-β-d-glucuronide (**44**) have been isolated eluting with methanol‒water (1:1) [34]. Nonetheless, apigenin-7-*O*-β-d-glucoside (**43**) were isolated from ethyl acetate extracts of aerial parts of *Thymus praecox* [35] and *Salvia macrosiphon* (eluent: methanol) [45] as two Lamiaceae species; moreover, apigenin-7-*O*-β-d-glucuronide (**44**) was purified from *n*-butanol fraction of *Erigeron multiradiatus* whole part (eluent: chloroform‒methanol 1:1) [54].

SLH was applied as the last chromatographic step in isolation of vitexin (**45**) (apigenin 8-*C*-glucoside) from hydroethanolic (60%) and petroleum ether extracts obtained from *Desmodium adscendens* [55] and *Indocalamus latifolius* [43] leaves, where methanol (20 to 100%) in water and pure methanol were used as eluting solvents, respectively.

Vitexin 2”-*O*-xyloside (**46**) and its iso-derivative namely isovitexin 2”-*O*-xyloside (**48**) have been formerly isolated from *Desmodium adscendens* leaf hydroethanolic (60%) extract utilizing methanol (20 to 100%) in water as eluent [55]; however, isovitexin (**47**) is apigenin-6-*C*-glucoside that has been isolated from ethanolic extract of *Croton zambesicus* leaf with ethyl acetate in methanol (10 to 100%) as eluting solvent in SLH [56].

Gohari et al. [45] isolated apigenin-7,4′-dimethyl ether (**49**) by finally exploiting SLH (eluent: methanol) from ethyl acetate extract fractionated from *Salvia macrosiphon* aerial part. From ethyl acetate extract of *Aquilaria sinensis* seeds, 7,4′-dimethylapigenin-5-*O*-xylosylglucoside (**50**) and 7,4′-dimethyl-5-*O*-glucosideflavonoide (**55**) eluting with methanol‒water (7:3), along with hydroxylgenkwanin (**51**), lethedoside A (**52**), 5,7-dihydroxyl-4′-methoxyflavone (**53**), and 7,3′-dimethyl-4′-hydroxyl-5-*O*-glucosideflavonoide (**54**) using methanol as eluent have been isolated and purified via SLH [57].

In another investigation, amentoflavone (**56**) was isolated from *Ginko biloba* leaf ethyl acetate extract by application of methanol as eluent [36]. Hispidulin (**57**) has been isolated from ethyl acetate extracts of sprig and flower of *Rosmarinus officinalis* [37] and *Chamaemelum nobile* [38] utilizing methanol‒water (2:1) and methanol‒dichloromethane (1:1), respectively. Root bark of *Morus alba* has been previously partitioned and its ethyl acetate extract was subjected to separation of their phytoconstituents, finally through methanol‒water (8:2) as eluent in SLH, 2 known flavones kuwanon T (**58**) and sanggenon J (**59**), in addition, two novel secondary metabolites sanggenon V (**60**) and sanggenon W (**61**) have been isolated accordingly [18].

Several other flavone derivatives have been also isolated and purified from different soluble-extracts of the species by SLH: hypoletin-7-*O*-β-d-xylopyranoside (**62**) from leaf ethyl acetate extract of *Thuja orientalis* (eluent: methanol) [58], galangin (**63**) from herb chloroform fraction of *Dalbergia cochinchinensis* (eluent: methanol‒dichloromethane 1:1) [12], 3′-geranyl-3-prenyl-2′,4′,5,7-tetrahydroxyflavone (**64**) from ethyl acetate extract of *Morus alba* root bark (eluent: methanol‒water 1:1) [19], pectolinarigenin (**65**) from chloroform fraction of *Cirsium Japonicum* aerial part [59], scutellarein-7-*O*-β-glucuronide (**66**) from *Erigeron multiradiatus n*-butanol aerial part extract [54], cirsimaritin (**67**), cirsilinelol (**68**), and eupatilin (**69**) from chloroform extract of aerial part of *Centaurea bruguierana* [60], eluting with chloroform‒methanol (1:1), and eupafolin (**70**) from ethyl acetate extract of *Chamaemelum nobile* aerial part (eluent: methanol‒dichloromethane 1:1) [38].

Tricin (**71**) is 5,7,4′-trihydroxy-3′,5′-dimethoxyflavone, comprising many valuable bio- and pharmacological properties [61], and it has been isolated from leaf ethyl acetate extract of *Sasa senanensis* (eluent: methanol‒water 6:4) [62], bract hydroethanolic (95%) fraction of *Zea mays* [63], and aqueous extract of *Phlomis bruguieri* aerial part (eluent: *n*-hexane‒methanol‒acetone 3:6:1) [40].

The application of SLH on hydroethanolic (95%) extract of *Zea mayes* bract has led to isolation of three tricin glucosides including tricin-5-*O*-β-d-glucopyranoside (**72**), tricin-7-*O*-β-d-glucopyranoside (**73**), and novel flavone namely tricin-7-*O*-[β-d-apifuranosyl (1→2)]-β-d-glucopyranoside (**74**) [63]. Tricin-7-*O*-β-d-glucopyranoside (**73**) has been furtherly isolated from two other Poaceae species *Avena sativa* [64] and *Indocalamus latifolius* [43], while a hydroethanolic (95%) fraction of bran and methanolic extract of leaf have been eluted by methanol in SLH column, respectively.

A new secondary metabolite 4′-methoxy-luteolin-7-phosphate (**75**) has been formerly isolated by hiring SLH (eluent: *n*-hexane‒methanol‒acetone 3:6:1) from aerial part aqueous extract of *Phlomis bruguieri* [40]. From an Asteraceae species *Santolina chamaecyparissus* nepetin (**76**) (eluent: methanol) was purified, where the dichloromethane extract of its aerial part was subjected to chromatographic procedure [65].

Isoetin (**77**) and its glycosylated analogous including isoetin-7-*O*-β-d-glucopyranosyl-2′-*O*-α-l-arabinopyranoside (**79**), isoetin-7-*O*-β-d-glucopyranosyl-2′-*O*-α-d-arabinopyranoside (**80**), and isoetin-7-*O*-β-d-glucopyranosyl-2′-*O*-α-d-xyloypyranoside (**81**), along with genkwanin (**82**) and genkwanin-4′-*O*-β-d-lutinoside (**83**), have been isolated and purified by applying SLH as the last separation stage (eluent: methanol) from methanol extract of *Taraxacum mongolicum* aerial part [22]. Notably, a novel flavone isoetin 2′-methyl ether (**78**) (5,7,4′,5′-tetrahydroxy-2′-methoxyflavone) has been isolated from *Bauhinia galpinii*, where the ethyl acetate extract of the leaf were applied by using acetone‒methanol (1:1) as eluting solvent via SLH [66].

By subjecting hydroethanolic (50%) extract of sugarcane top part of *Saccharum officinarum* to various chromatographic methods, albanin A (**84**), australone A (**85**), and 5′-geranyl-5,7,2′,4′-tetrahydroxy-flavone (**86**) have been finally isolated by exploiting chloroform‒methanol (1:1) and pure methanol as eluting solvents in SLH column [53]. In another study, methanolic extract obtained from whole part of *Dendrobium ellipsophyllum* were subjected to SLH (eluent: acetone) and chrysoeriol (**87**) was consequently isolated [23]. Xuan et al. [28] isolated 4′-methoxyflavone-6-*O*-β-d-glucopyranoside (**88**) for the first time in the nature from rhizome ethyl acetate extract of *Imperata cylindrica* by SLH (eluent: methanol), whereas 5-hydroxyflavone (**89**) was furtherly isolated from its petroleum ether extract by using dichloromethane‒methanol (1:1) as eluting mixture.

Three other flavones have also been isolated by SLH: texasin 7-*O*-β-d-glucopyranoside (**90**) from ethyl acetate extract of *Leptadenia pyrotechnica* aerial part [67], tilianin (**91**) from hydroethanolic (95%) extract of *Avena sativa* bran (eluent: methanol) [64], and 5-hydroxy-6,7,3′,4′-tetramethoxyflavone (**92**) from flower chloroform extract of *Citrus aurantium* (eluent: chloroform‒methanol 1:1) [68].

Moreover, 5 isoflavone derivatives have been isolated by SLH as the last separation procedure. Formononetin-7-*O*-β-d-glucosy1 [1,2,3,4,5,6] glucoside (**94**) and tectoridin (**95**) have been purified from ethyl acetate extract of *Maackia amurensis* bark (eluent: methanol‒water 6:4) [69], whilst formononetin (**93**) was extracted from *Aquilaria sinensis* stem ethyl acetate extract (eluent: methanol) [57]. Sphaerobioside (**96**) and a well-known isoflavone genistein (**97**) have been previously isolated by SLH eluting with methanol‒water (1:1) and methanol, respectively, from aqueous root fractions of *Cudrania tricuspidata* [25].

### 2.5. Flavonol Derivatives

SLH played a key role in isolation or purification of flavonoids specifically flavonol derivatives. The performed studies reported that SLH has been applied for isolation or purification of 79 different flavonol derivatives (**98**–**176**), while quercetin (**98**) with its analogous (**99**‒**129**), and kaempferol (**130**) and its analogous (**131**–**152**) were the most identified compound.

Quercetin (**98**) (3,3′,4′,5,7-pentahydroxyflavone, C_15_H_10_O_7_) is considered as one of the most beneficial flavonols and renowned for its antioxidant, anticancer, anti-inflammatory, and antiviral properties and endothelium-dependent vasodilation, and blood lipid-lowering effects [70,71,72,73]. SLH gel filtration chromatography has been able to isolate and purify quercetin (**98**). Nineteen studies reported the successful isolation of this compound from 19 diverse species by using SLH. According to the literature, it seems the ethyl acetate fractions of various plant species are the richest extracts in case of quercetin (**98**) content.

The calix part of *Fragaria ananassa* was solvent-solvent partitioned and finally by utilizing SLH (eluent: methanol‒water 6:4), quercetin (**98**) was isolated from the ethyl acetate extract [74]. The ethyl acetate extract of *Gynura divaricate* leaf has been furtherly subjected to isolate their major secondary metabolites, and the abovementioned compound was isolated by chloroform‒methanol (1:1) as an eluent system [75]. By eluting methanol through SLH column, quercetin (**98**) has been isolated from *Sarcopyramis bodinieri* ethyl acetate extract [76]. Quercetin (**98**) has also been isolated from ethyl acetate extracts of *Chionanthus retusus* flower (eluent: methanol‒water 8:2) [24], *Tamarix hohenackeri* aerial parts (eluent: methanol) [77], whole part of *Pteris vittata* (eluent: chloroform‒methanol 1:1) [78], *Populus davidiana* wood eluting with methanol‒water (3:1, 1:1, 1:3) [15], and from aerial part of *Halimodendron halodendron* (eluent: chloroform‒methanol 1:1) [79].

Several researchers isolated quercetin (**98**) from alcoholic extracts of different species via SLH as the last chromatographic step. Abou Zeid et al. [34] isolated this flavonol from hydroethanolic (70%) extract of *Brachychiton acerifolius* leaf eluting by methanol‒water (1:1) as eluent. In other phytochemical studies on *Byrsocarpus coccineus* (Connaraceae family) [80], *Juniperus chinensis* (Cupressaceae family) [81], and *Paulownia tomentosa* (Scrophulariaceae family) [14], this compound has been isolated from *n*-butanol fraction of the leaf, herb, and bark, while methanol, chloroform‒methanol (4:1), and methanol‒water (1:1) were applied as eluents, respectively. The methanolic extracts of *Cheilanthes tenuifolia* whole part [82] and *Taraxacum mongolicum* aerial part [22] have been exploited to isolate this phytochemical eluting with methanol (0 to 60%) in water and pure methanol, respectively.

Hydroalcoholic fractions of some species have been previously applied for isolation and purification of quercetin (**98**): hydro-methanolic (70%) extracts of leaf and aerial part of *Albizia amara* [83] and *Allium porrum* [84] eluting via methanol and methanol‒water (6:4), respectively, and hydro-ethanolic (50%) extract of sugarcane top part of *Saccharum officinarum* (eluent: chloroform‒methanol 1:1) [53]. Furthermore, this aglycone flavonol has been isolated from stem aqueous extract of *Bauhinia strychnifolia* using methanol as eluent in SLH gel filtration method [85]. Among all isolated quercetin derivatives (**99**‒**129**) by applying SLH, two aglycones, including 3-*O*-methylquercetin (**99**) and 3,3′-di-*O*-methylquercetin (**100**) have been isolated from the ethyl acetate extract of *Halimodendron halodendron* (Fabaceae) aerial part with mixture eluting solvents of chloroform‒methanol (1:1) [79].

Rutin (**101**) (syn. quercetin-3-*O*-*α*-rhamnosyl (1→6)-*β*-d-glucoside or 3′,4′,5,7-tetrahydroxy-flavone-3-rutinoside), as a well-renown dietary flavonoid, has been reported to possess several remarkable pharmacological benefits, such as in the treatment of Parkinson’s, and Alzheimer’s diseases, and myocardial infraction, along with anti-depressant, antihypertensive, anti-allergic, antioxidant, and anticancer properties [86,87,88]. However, this compound has been isolated by different methods, specifically solid-phase extraction and counter-current chromatography, and the size exclusion technique has also been applied to isolate this compound [86]. By utilization of SLH as the final purification phase, rutin (**101**) has been isolated from hydroethanolic (70%) and aqueous extracts of leaf and fruit of *Brachychiton acerifolius* [34] and *Cinnamomum zeylanicum* [89], respectively, by using methanol‒water (1:1), and from whole part methanolic fraction of *Cheilanthes tenuifolia* eluting with methanol (0 to 60%) in water [82].

Application of SLH has led to isolation of quercetin-3-*O*-β-6′’-(*p*-coumaroyl) glucopyranoside-3′-methyl ether (**102**) (syn. helichrysoside-3′-methyl ether) from ethanolic leaf extract of *Croton zambesicus* with chloroform (10 to 60%) in methanol as eluent [56]. Two glycosylated quercetin analogous quercetin 3-β-d-glucoside (**103**) and quercetin 3-*O*-α-arabinoside (**104**) have been isolated using *n*-butanol and ethyl acetate extracts of *Byrsocarpus coccineus* leaf, respectively, in which methanol was as eluting solvent [80].

From leaf ethyl acetate fractions of *Bauhinia galpinii* and *Dryopteris filix-mas* have been finally isolated quercetin-3-*O*-β-galactopyranoside (**105**) [66] and quercetin-3-*O*-α-l-rhamnopyranoside (**106**) [90] exploiting acetone‒methanol (1:1) and pure methanol as eluting solvent systems, respectively. Quercetin-3-*O*-α-l-rhamnopyranoside (**106**) has been furtherly isolated from aqueous and *n*-butanol extracts of flower and leaf of *Cinnamomum zeylanicum* [89] and *Curcuma longa* [91], respectively, by using methanol‒water as eluent mixture in SLH gel filtration column. The leaf *n*-butanol extract of *Ficus exasperate* was extracted and quercetin-3-*O*-β-rhamnoside (**107**) accordingly isolated via SLH (eluent: toluene‒ethanol 7:3) [92]. By utilization of methanol as eluting solvent through SLH column, quercetin-3-*O*-glucopyranoside (**108**) has been isolated from leaf methanolic and *n*-butanol extracts of *Indocalamus latifolius* [43] and *Sambucus ebulus* [93], respectively. Another glycosylated quercetin derivative namely quercetin-3-*O*-β-d-glucuronide (**109**) has been obtained by SLH from *n*-butanol, ethanol, and ethyl acetate extracts of leaf, leaf, and stem parts of *Curcuma longa* [91], *Eugenia jambos* [94], and *Nelumbo nucifera* [95], while methanol‒water (8:2), ethanol‒water (7:3), and methanol were applied as eluents, respectively.

In similar studies, quercetin-3-*O*-sambubioside (**110**) and quercetin-3-*O*-sophoroside (**112**) have been isolated from *n*-butanol and hydroethanolic (70%) extracts of *Eriobotrya japonica* [96] and *Poacynum hendersonii* [97] leaves, respectively, using methanol as solvent in SLH. Moreover, quercetin 3-*O*-gentiobioside (**111**) has been finally extracted by application of SLH from hydro-methanolic (70%) fraction of *Albizia amara* leaf [83] and *n*-butanol extract of *Oryza sativa* grain [98] eluting with methanol‒water.

Hydro-methanolic (70%) extracts have been previously obtained from *Albizia amara* leaf [83] and aerial part of *Allium porrum* [84], then by using methanol‒water as eluents, quercetin 3-*O*-*α*-rhamnopyranoside (**113**) has been isolated and identified. Three flavonol glucosides consist of quercetin-3-*O*-α-l-rhap-(1→2)-[α-l-rhap-(1→6)]-β-d-galactopyranoside (**114**), quercetin-3-*O*-α-l-rhap-(1→6)-β-d-galactopyranoside (**115**), and quercetin-3-*O*-α-l-rhap-(1→2)-α-l-rhamnopyranoside (**116**) have been isolated from *Curcuma longa* leaf *n*-butanol extracts, eluting with methanol‒water (1:1) [91]. Moreover, phytochemical analysis of hydro-methanolic (70%) extract of *Allium porrum* aerial part was finally led to isolation of quercetin-3-*O*-β-glucopyranosyl-7-*O*-α-rhamnopyranoside (**117**) and quercetin-4′-*O*-β-glucopyranoside (**118**), by using methanol‒water as eluting solvents with ratios of 2:8 and 4:6, respectively [84].

Shi et al. [22] isolated quercetin-3,7-di-*O*-β-d-di-glucopyranoside (**119**), quercetin-3′,4′,7-trimethyl ether (**120**), and quercetin-7-*O*-[β-d-glucopyranosyl(1→6)-β-d-glucopyranoside] (**121**) from methanolic extract of aerial part of *Taraxacum mongolicum* (eluent: methanol). From ethyl acetate extracts of two plant species belonging to Asteraceae family including *Onopordum alexandrinum* seed and *Tridax procumbens* whole part, quercimeritrin (syn. quercetin-7-*O*-glucoside) (**122**) [42] and quercetin-7-*O*-β-d-glucopyranosyl-(2→1)-α-l-rhamnose (**123**) [44] have been isolated, respectively; moreover, quercimeritrin (**122**) was isolated from aqueous extract of *Cudrania tricuspidata* bark eluting with methanol‒water (1:1) [25].

SLH gel filtration method has also been used for isolation and purification of other glycosylated quercetin derivatives: dihydroquercetin 7-*O*-β-d-glucoside (**124**) from leaf *n*-butanol extract of *Curcuma longa* (eluent: methanol‒water 1:1) [91], quercetrin (syn. quercetin 3-*O*-rhamnoside) (**125**) from leaf butanol extract of *Camellia japonica* eluting with chloroform‒methanol (1:1) [99], and isoquercetin (syn. quercetin 3-β-*O*-glucoside) (**126**) from ethyl acetate fraction of *Dorema glabrum* aerial part (eluent: methanol‒water 8:2) [100].

Quercitrin (syn. quercetin-3-rhamnoside) (**127**) has been formerly isolated from *Thuja orientalis* leaf ethyl acetate extract [58], bran hydroethanolic (95%) extract of *Avena sativa* [64], and *Eriobotrya japonica* leaf *n*-butanol extract [96], whilst methanol was used as eluting solvent. Isoquercitrin (syn. quercetin 3-*O*-β-d-glucopyranoside) (**128**) has been previously isolated and identified from *n*-butanol extracts of *Phyllanthus reticulatus* leaf [101] and *Juniperus chinensis* herb [81], using methanol‒water (1:1) and methanol for eluting, respectively. This compound has also been isolated from hydroethanolic (70%) and ethyl acetate fractions yielded from leaves of *Poacynum hendersonii* [97] and *Thuja orientalis* [58], respectively, eluting with methanol. Hiring SLH eluting with toluene‒ethanol (7:3) has been concluded to isolate isoquercitrin-6-*O*-4-hydroxybenzoate (**129**) from *n*-butanol extract of *Ficus exasperate* leaf [92].

Kaempferol (**130**) (3,4′,5,1-tetrahydroxyflavoune) is an aglycone flavonol which is naturally occurred in many plants’ parts through the phenylpropanoid pathway [102,103]. Pharmacological and biological activities of this nutraceutical compound have been extensively studied and reported to possess significant antiproliferative, cytotoxicity, anti-inflammatory, antioxidant, and antidiabetic activities [104,105,106,107,108,109].

This valuable compound has been isolated from 11 different plant species by employing SLH as the last chromatographic step. Ethyl acetate extracts might be considered as the richest fractions in kaempferol (**130**) content: from *Fragaria ananassa* calyx (eluent: acetone‒water 2:1) [74], *Gynura divaricate* leaf (eluent: chloroform‒methanol 1:1) [75], *Gingko biloba* leaf (eluent: methanol) [36], *Chionanthus retusus* flower (eluent: methanol‒water 7:3) [24], *Populus davidiana* wood (methanol‒water 3:1, 1:1, 1:3) [15], and *Leptadenia pyrotechnica* aerial parts [67]. Kaempferol (**130**) has been furtherly isolated from hydro-methanolic (70%) extracts of *Albizia amara* leaf [83] and *Allium porrum* aerial part [84] eluting with methanol and methanol‒water (8:2), respectively. From leaf hydroethanolic (70%) extract of *Brachychiton acerifolius* applying methanol‒water (1:1) as eluting solvent [34], and aqueous fractions of *Zygophyllum dumosum* shoot [110] and *Cudrania tricuspidata* bark [25] with methanol through SLH, kaempferol (**130**) have been also isolated.

Among 23 kaempferol derivatives (**131**–**152**), only 7,4′-dimethoxykaempferol (**131**) has been isolated as aglycone analogue from aerial part ethyl acetate extract of *Tamarix hohenackeri* (Tamaricaceae family) using methanol as eluent in SLH column [77]. The leaf ethanolic and ethyl acetate extracts of *Croton zambesicus* and *Gingko biloba* have been previously subjected to various chromatographic methods, and tiliroside (syn. kaempferol-3-*O*-β-6′’(*p*-coumaroyl)-glucopyranoside) (**132**) [56] and kaempferol 3-*O*-rhamnopyranoside (**133**) [36] have been accordingly isolated via chloroform‒methanol (9:1) and methanol as SLH eluent, respectively.

Among all the isolated secondary metabolites from leaf *n*-butanol extract of *Curcuma longa*, kaempferol-3-*O*-α-l-rhamnopyranoside (**134**) has been identified as a glycosylated flavonol exploiting methanol‒water (8:2) for eluting of samples in SLH [91]. Kaempferin (syn. afzelin, Kaempferol-3-rhamnoside) (**135**) has been previously isolated from two plant species of *Eriobotrya japonica* [96] and *Thuja orientalis* [58], whereas their leaves *n*-butanol and ethyl acetate extracts were chromatographed by SLH with methanol, respectively. Methanol has been used as eluting solvent in isolation and purification of Kaempferol-3-rutinoside (**136**) from *Sideroxylon foetidissimum* leaf petroleum ether extract [111] and kaempferol 3-*O*-α-arabinoside (**137**) from ethanolic fraction of *Opuntia dilleniid* flower [112].

Kaouadji et al. [113] isolated kaempferol 3-*O*-α-l-(2-*E*-*p*-coumaroyl rhamnopyranoside) (**138**) and kaempferol 3-*O*-α-l-(2-*Z*-*p*-coumaroyl rhamnopyranoside) (**139**) from ethyl acetate extract of buds of *Platanus acerifolia* by SLH (eluent: methanol). In a phytochemical investigation carried out on *Nelumbo nucifera*, the ethyl acetate extract of stem by utilization of methanol in SLH gel filtration column kaempferol 3-*O*-α-l-rhamnopyranosyl-(1→6)-β-d-glucopyranoside (**140**), kaempferol 3-*O*-β-(2″-*O*-α-rhamnosyl)-glucuronide (**141**), and kaempferol 3-*O*-α-l-rhamnopyranosyl-(1→2)-β-d-glucopyranoside (**142**), and kaempferol 3-*O*-β-d-glucuronopyranoside (**143**) have been isolated and purified [95].

SLH has been able to isolate astragalin (syn. kaempferol 3-*O*-β-d-glucopyranoside) (**144**) from five plant species. Hydro-methanolic (70%) and hydroethanolic (95%) extracts of *Allium porrum* aerial part [84] and bran part of *Avena sativa* [64] have been applied to isolate astragalin (**144**) applying methanol‒water (6:4) and methanol as eluting solvent, respectively. Furthermore, aerial parts ethyl acetate extracts of *Leptadenia pyrotechnica* [67] and *Dorema glabrum* [100], and *Fragaria ananassa* calyx (eluent: acetone‒water 7:3) [74] comprised the aforementioned compound.

From aerial part ethyl acetate extract of *Leptadenia pyrotechnica* kaempferol-3-*O*-α-l-rhamnopyranosyl (1″′→6″)-*O*-β-d-glucopyranoside (**145**) and kaempferol-3-*O*-β-d-glucopyranosyl (1″′→6″)-*O*-β-d-glucopyranoside (**146**) [67], whereas kaempferol 3-*O*-(3″-*E-p*-coumaroyl)-α-l-rhamnopyranoside (**147**) and kaempferol 3-*O*-(2″*-O-E-p-*coumaroyl)-β-d-glucopyranoside (**148**) were also isolated and identified from bran hydroethanolic (95%) extract of *Avena sativa* (eluent: methanol) [64].

Rayyan et al. [16] reported isolation of a novel kaempferol glucoside, namely 8-methoxykaempferol 3-*O*-(6″-malonyl-β-glucopyranoside) (**149**) from hydro-methanolic (80%) extract of leaf and flower parts of *Crataegus* spp. (Hawthorn) by increasing ratio of methanol (40 to 70%) in water using SLH.

According to previously performed phytochemical studies, three other glycosylated kaempferol derivatives have been furtherly isolated and purified by SLH gel filtration: kaempferol 7-*O*-glucoside (**150**) from seed ethyl acetate extract of *Onopordum alexandrinum* (eluent: methanol‒water 9:1) [42], kaempferol 7-*O*-β-glucopyranoside (**151**) from hydro-methanolic (70%) fraction of *Allium porrum* aerial part (eluent: methanol‒water 6:4) [84], and kaempferol 7-*O*-α-l-rhamnopyranoside (**152**) from bran hydro-ethanolic (95%) extract of *Avena sativa* (eluent: methanol) [64].

Isorhamnetin (**153**) has been isolated by using SLH eluting with methanol‒water (8:2) from hydro-methanolic (70%) extract of *Allium porrum* aerial parts [84]. Three isorhamnetin glucosides consist of isorhamnetin 3-*O*-β-d-rutinoside (**154**) from aerial part ethyl acetate extract of *Halimodendron halodendron* (eluent: chloroform‒methanol 1:1) [79] and flower ethanolic fraction of *Opuntia dillenii* (eluent: methanol) [112], isorhamnetin 3-*O*-monoglucoside (**155**) from *n*-butanol extract of *Sambucus ebulus* leaf (eluent: methanol) [93], along with isorhamnetin 3-*O*-β-d-glucopyranoside (**156**) from *Dorema glabrum* aerial part ethyl acetate extract (eluent: methanol‒water 8:2) [100].

Exploiting SLH by eluting acetone‒methanol (1:1), myricetin (syn. 3,5,7,3′,4′,5′-hexahydroxyflavone) (**157**) and myricetin-3-*O*-β-galactopyranoside (**160**) have been isolated from ethyl acetate extract of *Bauhinia galpinii* leaf [66]. Moreover, from stamen ethyl acetate, a fraction of *Nelumbo nucifera*, myricetin 3′,5′-dimethylether 3-*O*-β-d-glucopyranoside (**158**) (eluent: methanol) [114], and a novel secondary metabolite myricetin 7-methylether 3-*O*-xylopyranosylsyl-(1→2)-α-rhamnopyranoside (**159**) have been previously isolated and identified from *Eugenia jambos* ethanolic extract of the leaf (eluent: ethanol‒water 3:7) [94].

By eluting methanol‒water and pure methanol through SLH column, myricitrin (syn. myricetin 3-*O*-α-rhamnopyranoside) (**161**) has been isolated from hydro-methanolic (70%) and ethyl acetate extracts of *Albizia amara* [83] and *Thuja orientalis* [58], respectively. Another study reported SLH was able to isolate penduletin (**162**) and chrysosplenol D (**163**) from aerial part methanolic extract of *Plectranthus cylindraceus* [115].

More flavonol derivatives have also been isolated and purified from ethyl acetate extracts of diverse plant species: sexangularetin (**164**) from calyx of *Fragaria ananassa* eluting with methanol‒water (4:1) [74], a new natural product brassicin-4′-*O*-β-d-glucopyranoside (**165**) via increasing acetone ratio (33 to 100%) in water from *Oryza sativa* spp. *japonica* grain [116], 5,7,3′-trimethyl-4′-methoxyl-3-*O*-β-d-flavonoid glucoside (**166**) and 8,3′-dihydroxyl-3,7,4′-trimethoxy-6-*O*-β-d-flavonoid glucoside (**167**) from whole part of *Tridax procumbens* [44], a novel phytochemical ptevon-3-d-glucoside (**168**) from *Pterocarpus indicus* leaf (eluent: dichloromethane‒methanol 1:1) [117], leonurusoide E (**170**) from *Leonurus japonicus* eluting with methanol‒water (4:6) [118], dillenetin (**172**) from *Tamarix hohenackeri* aerial part (eluent: methanol‒water) [77], and tamarixetin 3-*O*-rhamnopyranoside (**175**) from *Firmiana simplex* stem bark (eluent: methanol) [119].

Furthermore, sophoflavescenol (**169**) from root dichloromethane extract of *Sophora flavescens* (eluent: dichloromethane‒methanol) [120], 5,4′-dihydroxyflavone-3,6-di-*O*-β-d-glucoside-7-*O*-β-d-glucuronide (**171**) from *Carthamus tinctorius* aqueous flower fraction (eluent: water) [121], 7-hydroxy-6-methoxyflavone (**173**) from herb chloroform extract of *Dalbergia cochinchinensis* (dichloromethane‒methanol 1:1) [12], 3-*O*-demethyldigicitrin (**174**) from ethanolic extract of *Athrixia phylicoides* aerial part (eluent: methanol), and artemitin (**176**) from methanolic fraction of *Taraxacum mongolicum* [22] have been formerly isolated and purified by application of SLH as the final separation step.

### 2.6. Homoisoflavonoid Derivatives

Homoisoflavonoids are naturally occurred mostly in Asparagaceae and Fabaceae families. Several research groups reported their antimicrobial, antidiabetic, cytotoxic, anticancer, anti-inflammatory, antimutagenic, etc. properties [122,123].

In a previous phytochemical investigation performed on hydroethanolic (60%) extract obtained from the rhizome of *Polygonatum odoratum* (Asparagaceae family), SLH gel filtration column eluted by acetonitrile‒methanol (1:1) was applied to isolate 10 homoisoflavonoids (**177**‒**186**) including three novel natural products of (3*R*)-5,7-dihydroxy-8-methyl-3-(2′,4′-dihydroxybenzyl)-chroman-4-one (**177**), (3*R*)-5,7-dihydroxy-8-methyl-3-(4′-hydroxybenzyl)-chroman-4-one (**181**), and (3*R*)-5,7-dihydroxy-3-(2′-hydroxy-4′-methoxybenzyl)-chroman-4-one (**182**); whereas (3*R*)-5,7-dihydroxy-6-methoxy-8-methyl-3-(2′,4′-dihydroxybenzyl)-chroman-4-one (**178**), (3*R*)-5,7-dihydroxy-3-(4′-hydroxybenzyl)-chroman-4-one (**179**), (3*R*)-5,7-dihydroxy-8-methoxy-3-(2′-hydroxy-4′-methoxybenzyl)-chroman-4-one (**180**), (3*R*)-5,7-dihydroxy-6-methyl-3-(4′-hydroxybenzyl)-chroman-4-one (**183**), (3*R*)-5,7-dihydroxy-6-methyl-8-methoxy-3-(4′-hydroxybenzyl)-chroman-4-one (**184**), (3*R*)-5,7-dihydroxy-6,8-dimethyl-3-(4′-hydroxybenzyl)-chroman-4-one (**185**), and (3*R*)-5,7-dihydroxy-6-methyl-8-methoxy-3-(4′-methoxybenzyl)-chroman-4-one (**186**) have been isolated as known homoisoflavonoid analogous [124].

### 2.7. Proanthocyanidins

Proanthocyanidins are condensed tannins, considered as the end product of flavonoid biosynthetic pathway with various health characteristic advantages, for instance, antioxidant, anticancer, antidiabetic, neuroprotective, and antimicrobial potencies, and the treatment of cardiovascular disease [125,126].

Utilization of SLH has led to isolate 3 proanthocyanidin derivatives from two Lauraceae species. Cinnamtannin B1 (syn. epicatechin-(2β→O-7,4β→8)-epicatechin-(4β→8) epicatechin) (**187**) has been isolated from herb ethyl acetate extract of *Lindera glauca* [127] and aqueous fraction of *Cinnamomum zeylanicum* fruit [89] by hiring methanol‒water as eluting solvent. Huh et al. [127] reported isolation of two proanthocyanidins of cinnamtannin D1 (**188**) and procyanidin A1 (**189**) from herb ethyl acetate of *Lindera glauca* by application of SLH eluting with methanol‒water 1:1, and 1:1 to 5:1 of this solvent mixture, respectively.

## 3. Conclusions

Nowadays, new separation techniques have been established to facilitate analysis of natural products. The isolation and purification of flavonoids as one of the most valuable natural compounds have been carried out by applying several classical and recently developed methods. SLH as a type of size-exclusion chromatography (syn. gel filtration) has been broadly used in isolation or purification of flavonoid analogous.

The present context overviewed the role of SLH in isolation or purification of flavonoids as the final chromatographic step. This review for the first time provides valuable information about the classification of isolated flavonoids, plant families and species, the used plant parts and extracts, and applied eluents utilized in SLH gel filtration chromatography.

In brief, SLH has been able to isolate or purify 189 flavonoids categorized in seven classes, mostly comprised of 79 flavonols and 63 flavones. Notably, six flavones (**60**,**61**,**74**,**75**,**78**,**88**), four flavonols (**149**,**159**,**165**,**168**), three homoisoflavonoids (**177**,**181**,**182**), one flavanone (**8**), a flavanol (**26**), and an isoflavanol (**29**) have been isolated as the novel secondary metabolites in nature. The Asteraceae possessing 22 flavone and 13 flavonol derivatives has been documented as the richest plant family, which was subjected to finally SLH for isolation or purification of their flavonoids. Homoisoflavonoids and proanthocyanidins have been only isolated from the Asparagaceae and Lauraceae families, respectively. Furthermore, methanol and methanol‒water has been majorly applied as eluents to perform the separation process.

In general, the flavonoids have mainly been isolated or purified from hydro-alcoholic and ethyl acetate extracts. The flavan and isoflavan compounds have been isolated from chloroform extracts. Eleven flavanones (from totally 18), were isolated from ethyl acetate extracts, while methanol fractions were applied for isolation of four derivatives. Aqueous extracts were the richest in flavanol compounds (six of eight). Thirty flavones from ethyl acetate extracts, 17 derivatives from hydro-ethanolic (50 to 95%), and 11 compounds from methanolic extracts have been isolated or purified.

From totally 79 flavonols, combination of methanol and water have majorly been used for isolation or purification of the most flavonols, where 19 compounds from different ratios, and 15 derivatives from the equivalent ratio (1:1) of this mixture were isolated. Hydro-ethanolic (60%) extract was applied for isolation of all homoisoflavonoids, while all four proanthocyanidins were isolated from ethyl acetate extracts.

## Figures and Tables

**Table 1:** Abundant of flavonoids isolated/purified by using different eluents on Sephadex^®^ LH-20.

Flavonoid Classes	Eluent Systems																
	A	B	C	D	E	F	G	H	I	J	K	L	M	N	O	P	Q
Flavan & Isoflavan							2										
Flavanone	1						5						4	9			
Flavanol & Isoflavanol													2	7			
Flavone & Isoflavone	1		1				8	9		1	2		28	22	4	2	
Flavonol			3	3			3	8				1	37	26			2
Homoisoflavonoids					10												
Proanthocyanidin														3			

A: acetone; B: acetone-H_2_O; C: acetone-MeOH; D: acetone-H_2_O; E: acetonitrile‒MeOH; F: CH_2_Cl_2_; G: CH_2_Cl_2_–MeOH; H: CHCl_3_–MeOH; I: EtOAc; J: EtOAc‒MeOH; K: EtOH-H_2_O; L: H_2_O; M: MeOH; N: MeOH-H_2_O; O: *n*-hexane‒MeOH‒acetone; P: *n*-hexane-EtOAc; EtOAc; EtOAc–MeOH; Q: toluene‒EtOH.

## Supplementary Materials

The following are available online at https://www.mdpi.com/1420-3049/25/18/4146/s1, Table S1. Isolated or purified flavonoid derivatives by utilizing Sephadex^®^ LH-20 from diverse plant families.
