# Supplementary material for: Sephadex® LH-20, Isolation, and Purification of Flavonoids from Plant Species: A Comprehensive Review
Source: Molecules. 2020 Sep 10;25(18):4146. doi: 10.3390/molecules25184146 (PMC7570886; doi:10.3390/molecules25184146)
Supplement: Supplementary file 1 [file molecules-25-04146-s001.pdf]

Table S1. Isolated or purified flavonoid derivatives by utilizing Sephadex® LH-20 from diverse plant families

No.	Name	Classification	Plant species	Family	Subjected extract/plant part	Solvent used in Sephadex® LH-20	Ref.
1	6,4′-dihydroxy-7-methoxy-flavan	flavan	*Dalbergia cochinchinensis*	Fabaceae	$CHCl_3$/H	CH_2Cl_2–MeOH (1:1)	[10]
2	mucronulatol	isoflavan	*Dalbergia cochinchinensis*	Fabaceae	$CHCl_3$/H	CH_2Cl_2–MeOH (1:1)	[10]
3	dihydrowogonin	flavanone	*Chenopodium procerum*	Chenopodiaceae	CH_2Cl_2/AP	MeOH	[11]
4	naringenin		*Paulownia tomentosa*	Scrophulariaceae	*n*-BuOH/B	MeOH–H_2O (1:1, 1:3)	[40]
			Dalbergia cochinchinensis	Fabaceae	$CHCl_3$/H	CH_2Cl_2–MeOH (1:1)	[10]
			Populus davidiana	Salicaceae	EtOAc/W	MeOH–H_2O (3:1, 1:1, 1:3)	[13]
5	naringenin 7-*O*-β-glucopyranoside (syn. prunin)		*Crataegus* spp. (Hawthorn)	Rosaceae	MeOH (80%)/L, Fl	MeOH (40-70%)–H_2O	[14]
6	sanggenol Q		*Morus alba*	Moraceae	EtOAc/RB	MeOH–H_2O (8:2)	[15]
7	sanggenon F		*Morus alba*	Moraceae	EtOAc/RB	MeOH–H_2O (8:2)	[15]
8	sanggenon U		*Morus alba*	Moraceae	EtOAc/RB	MeOH–H_2O (8:2)	[16]
9	kuwanon E		*Morus alba*	Moraceae	EtOAc/RB	MeOH–H_2O (8:2)	[16]
10	euchrenone a7		*Morus alba*	Moraceae	EtOAc/RB	MeOH–H_2O (7:3)	[16]
11	sanggenon J		*Morus alba*	Moraceae	EtOAc/RB	MeOH	[17]
12	sanggenon F		*Morus alba*	Moraceae	EtOAc/RB	MeOH–H_2O (1:1)	[17]
13	sanggenol A		*Morus alba*	Moraceae	EtOAc/RB	MeOH–H_2O (1:1)	[17]
14	pinocembrin		*Corema album*	Ericaceae	EtOAc/L	CH_2Cl_2–MeOH (1:1)	[18]
			Dalbergia cochinchinensis	Fabaceae	PE/H	CH_2Cl_2–MeOH (1:1)	[10]
15	liquiritigenin		*Dalbergia cochinchinensis*	Fabaceae	$CHCl_3$/H	CH_2Cl_2–MeOH (1:1)	[10]
16	alpinetin		*Dalbergia cochinchinensis*	Fabaceae	$CHCl_3$/H	CH_2Cl_2–MeOH (1:1)	[10]
17	7,8-dihydroxyflavanone		*Dalbergia cochinchinensis*	Fabaceae	EtOAc/H	CH_2Cl_2–MeOH (1:1)	[10]
18	hesperidin		*Taraxacum mongolicum*	Asteraceae	MeOH/AP	MeOH	[21]
19	4′,5,7-trihydroxy-3′-methoxyflavanone		*Taraxacum mongolicum*	Asteraceae	MeOH/AP	MeOH	[21]
20	(2*S*)-homoeriodictyol		*Dendrobium ellipsophyllum*	Orchidaceae	MeOH/WP	acetone	[22]
21	aromadendrin	flavanol	*Chionanthus retusus*	Oleaceae	EtOAc/Fl	MeOH–H_2O (8:2)	[23]
			Cudrania tricuspidata	Moraceae	Aqueous/B	MeOH	[24]
22	dihydroquercetin (syn. taxifolin)		*Chionanthus retusus*	Oleaceae	EtOAc/Fl	MeOH–H_2O (8:2)	[23]
			Cudrania tricuspidata	Moraceae	Aqueous/B	MeOH–H_2O (1:1)	[24]
23	dihydroquercetin-7-*O*-β-D-glucoside (syn. taxifolin 7-glucoside)		*Cudrania tricuspidata*	Moraceae	Aqueous/B	MeOH–H_2O (1:1)	[24]

24	6-*p*-hydroxybenzyl taxifolin-7-*O*-β-D-glucoside		*Cudrania tricuspidata*	Moraceae	Aqueous/B	MeOH–H_2O (1:1)	[24]
25	2,3-trans-dihydromorin		*Morus alba*	Moraceae	EtOAc/RB	MeOH	[17]
26	(2R,3S)-guibourtinidol-3-*O*-α-D-apiofuranosyl-(1→6)-*O*-β-D-glucopyranoside		*Morus alba*	Moraceae	*n*-BuOH/RB	MeOH–H_2O (3:2)	[25]
27	gericudranin E		*Cudrania tricuspidata*	Moraceae	Aqueous/B	MeOH–H_2O (1:1)	[24]
28	gericudranin C		*Cudrania tricuspidata*	Moraceae	Aqueous/B	MeOH–H_2O (1:1)	[24]
29	kotstrigoisoflavanol	isoflavanol	*Kotschya strigosa*	Fabaceae	MeOH/Fr	nd	[26]
30	flavone	flavone	*Imperata cylindrica*	Poaceae	EtOAc/Rh	CH_2Cl_2–MeOH (1:1)	[27]
31	3′,4′,7-trihydroxyflavone		*Albizzia julibrissin*	Fabaceae	EtOAc/SB	MeOH	[28]
32	5-hydroxy-6,7,8,3′,4′,5′-hexamethoxyflavon-3-ol		*Athrixia phylicoides*	Asteraceae	EtOH/AP	MeOH	[29]
33	4′-hydroxy-5-methoxyflavone		*Imperata cylindrica*	Poaceae	EtOAc/Rh	CH_2Cl_2–MeOH (1:1)	[27]
34	luteolin		*Brachychiton acerifolius*	Malvaceae	EtOH (70%)/L	MeOH–H_2O (1:1)	[33]
			Thymus praecox	Lamiaceae	EtOAc/AP	nd	[34]
			Gingko biloba	Ginkgoaceae	EtOAc/L	MeOH	[35]
			Rosmarinus officinalis	Lamiaceae	EtOAc/Sp	MeOH–H_2O (1:1)	[36]
			Chamaemelum nobile	Asteraceae	EtOAc/Fl	MeOH–CH_2Cl_2 (1:1)	[37]
			Phlomis bruguieri	Lamiaceae	Aqueous/AP	*n*-hexane–MeOH–acetone (3:6:1)	[39]
			Taraxacum mongolicum	Asteraceae	MeOH/AP	MeOH	[21]
			Populus tomentosa	Salicaceae	*n*-BuOH/X	MeOH–H_2O (1:1, 1:3)	[40]
			Populus davidiana	Salicaceae	EtOAc/W	MeOH–H_2O (3:1, 1:1, 1:3)	[13]
			Solenostemon monostachys	Lamiaceae	EtOAc/AP	*n*-hexan–EtOAc (3:7, 2:8, 1:9), EtOAc (100%), EtOAc–MeOH (1:9, 2:8, 4:6, 5:5)	[38]
			Dendrobium ellipsophyllum	Orchidaceae	MeOH/WP	acetone	[22]
35	7-methoxy luteolin		*Onopordum alexandrinum*	Asteraceae	EtOAc/Se	MeOH–H_2O (9:1)	[41]
36	orientin (syn. luteolin 8-*C*-glucoside)		*Indocalamus latifolius*	Poaceae	PE/L	MeOH	[42]
37	luteolin-7-*O*-β-D-glucoside (syn. cynaroside)		*Tilia rubra*	Tiliaceae	MeOH (80%)/L	MeOH–H_2O (8:2)	[45]
			Tridax procumbens	Asteraceae	EtOAc/WP	nd	[43]
			Olea europaea	Oleaceae	EtOH (50%)/L	EtOH (0-50%)–H_2O	[45]
			Salvia macrosiphon	Lamiaceae	EtOAc/AP	MeOH	[45]
			Citrus unshiu	Rutaceae	$CHCl_3$/P	MeOH–H_2O (1:1)	[47]
			Brachychiton acerifolius	Malvaceae	EtOH (70%)/L	MeOH–H_2O (1:1)	[33]

38	luteolin-7-*O*-β-D-galactopyranoside	*Taraxacum mongolicum*	Asteraceae	MeOH/AP	MeOH	[21]
39	luteolin-7-*O*-β-D-glucopyranoside	*Taraxacum mongolicum*	Asteraceae	MeOH/AP	MeOH	[21]
40	luteolin-4′-*O*-β-glucoside	*Olea europaea*	Oleaceae	EtOH (50%)/L	EtOH (0-50%)–H_2O	[45]
41	apigenin	*Brachychiton acerifolius*	Malvaceae	EtOH (70%)/L	MeOH–H_2O (1:1)	[33]
		Chamaemelum nobile	Asteraceae	EtOAc/Fl	CH_2Cl_2–MeOH (1:1)	[37]
		Phlomis bruguieri	Lamiaceae	Aqueous/AP	*n*-hexane–MeOH–acetone (3:6:1)	[39]
		Populus tomentosa	Salicaceae	*n*-BuOH/X	MeOH–H_2O (1:1, 1:3)	[40]
		Solenostemon monostachys	Lamiaceae	EtOAc/AP	*n*-hexane–EtOAc (3:7, 2:8, 1:9), EtOAc (100%), EtOAc–MeOH (1:9, 2:8, 4:6, 5:5)	[38]
		Saccharum officinarum	Poaceae	EtOH (50%)/ ST	$CHCl_3$–MeOH (1:1)	[53]
42	apigenin-7-*O*-*α*-rhamnosyl (1→2)-β-*D*-glucuronide	*Brachychiton acerifolius*	Malvaceae	EtOH (70%)/L	MeOH–H_2O (1:1)	[33]
43	apigenin-7-*O*-β-D-glucoside	*Brachychiton acerifolius*	Malvaceae	EtOH (70%)/L	MeOH–H_2O (1:1)	[33]
		Thymus praecox	Lamiaceae	EtOAc/AP	nd	[34]
		Salvia macrosiphon	Lamiaceae	EtOAc/AP	MeOH	[44]
44	apigenin-7-*O*-β-D-glucuronide	*Brachychiton acerifolius*	Malvaceae	EtOH (70%)/L	MeOH–H_2O (1:1)	[33]
		Erigeron multiradiatus	Asteraceae	*n*-BuOH/WP	$CHCl_3$–MeOH (1:1)	[54]
45	apigenin 8-*C*-glucoside (syn. vitexin)	*Desmodium adscendens*	Fabaceae	EtOH (60%)/L	MeOH (20–100%)–H_2O	[55]
		Indocalamus latifolius	Poaceae	PE/L	MeOH	[42]
46	vitexin 2″-*O*-xyloside	*Desmodium adscendens*	Fabaceae	EtOH (60%)/L	MeOH (20–100%)–H_2O	[55]
47	apigenin-6-*C*-glucoside (syn. isovitexin)	*Croton zambesicus*	Euphorbiaceae	EtOH/L	EtOAc–MeOH (10–100%)	[56]
48	isovitexin 2″-*O*-xyloside	*Desmodium adscendens*	Fabaceae	EtOH (60%)/L	MeOH (20–100%)–H_2O	[55]
49	apigenin-7,4′-dimethyl ether	*Salvia macrosiphon*	Lamiaceae	EtOAc/AP	MeOH	[44]
50	7,4′-dimethylapigenin-5-*O*-xylosylglucoside	*Aquilaria sinensis*	Thymelaeaceae	EtOAc/S	MeOH–H_2O (7:3)	[57]
51	hydroxylgenkwanin	*Aquilaria sinensis*	Thymelaeaceae	EtOAc/S	MeOH	[57]
52	lethedoside A	*Aquilaria sinensis*	Thymelaeaceae	EtOAc/S	MeOH	[57]
53	5,7-dihydroxyl-4′-methoxyflavone	*Aquilaria sinensis*	Thymelaeaceae	EtOAc/S	MeOH	[57]
54	7,3′-dimethyl-4′-hydroxyl-5-*O*-glucosideflavonoide	*Aquilaria sinensis*	Thymelaeaceae	EtOAc/S	MeOH	[55]
55	7,4′-dimethyl-5-*O*-glucosideflavonoide	*Aquilaria sinensis*	Thymelaeaceae	EtOAc/S	MeOH–H_2O (7:3)	[57]

56	amentoflavone	*Gingko biloba*	Ginkgoaceae	EtOAc/L	MeOH	[35]
57	hispidulin	*Rosmarinus officinalis*	Lamiaceae	EtOAc/Sp	MeOH–H_2O (2:1)	[36]
		Chamaemelum nobile	Asteraceae	EtOAc/Fl	CH_2Cl_2–MeOH (1:1)	[37]
58	kuwanon T	*Morus alba*	Moraceae	EtOAc/RB	MeOH–H_2O (8:2)	[15]
59	sanggenon J	*Morus alba*	Moraceae	EtOAc/RB	MeOH–H_2O (8:2)	[16]
60	sanggenon V	*Morus alba*	Moraceae	EtOAc/RB	MeOH–H_2O (8:2)	[16]
61	sanggenon W	*Morus alba*	Moraceae	EtOAc/RB	MeOH–H_2O (8:2)	[16]
62	hypoletin-7-*O*-β-D-xylopyranoside	*Thuja orientalis*	Cupressaceae	EtOAc/L	MeOH	[58]
63	galangin	*Dalbergia cochinchinensis*	Fabaceae	$CHCl_3$/H	CH_2CI_2–MeOH (1:1)	[10]
64	3′-geranyl-3-prenyl-2′,4′,5,7-tetrahydroxyflavone	*Morus alba*	Moraceae	EtOAc/RB	MeOH–H_2O (1:1)	[17]
65	pectolinarigenin	*Cirsium Japonicum*	Asteraceae	$CHCI_3$/AP	$CHCI_3$–MeOH (1:1)	[59]
66	scutellarein-7-*O*-β-glucuronide	*Erigeron multiradiatus*	Asteraceae	*n*-BuOH/WP	$CHCI_3$–MeOH (1:1)	[54]
67	cirsimaritin	*Centaurea bruguierana*	Asteraceae	$CHCI_3$/AP	$CHCI_3$–MeOH (1:1)	[60]
68	cirsilinelol	*Centaurea bruguierana*	Asteraceae	$CHCI_3$/AP	$CHCI_3$–MeOH (1:1)	[60]
69	eupatilin	*Centaurea bruguierana*	Asteraceae	$CHCI_3$/AP	$CHCI_3$–MeOH (1:1)	[60]
70	eupafolin	*Chamaemelum nobile*	Asteraceae	EtOAc/Fl	CH_2Cl_2–MeOH (1:1)	[37]
71	tricin	*Sasa senanensis*	Poaceae	EtOAc/L	MeOH–H_2O (6:4)	[62]
		Zea mays	Poaceae	EtOH (95%)/Br	nd	[63]
		Phlomis bruguieri	Lamiaceae	Aqueous/AP	*n*-hexane–MeOH–acetone (3:6:1)	[39]
72	tricin-5-*O*-β-D-glucopyranoside	*Zea mays*	Poaceae	EtOH (95%)/Br	nd	[63]
73	tricin-7-*O*-β-D-glucopyranoside	*Zea mays*	Poaceae	EtOH (95%)/Br	nd	[63]
		Avena sativa	Poaceae	EtOH (95%)/bran	MeOH	[64]
		Indocalamus latifolius	Poaceae	MeOH/L	MeOH	[42]
74	tricin-7-*O*-[β-D-apifuranosyl (1→2)]-β-D-glucopyranoside	*Zea mays*	Poaceae	EtOH (95%)/Br	nd	[63]
75	4′-methoxy-luteolin-7-phosphate	*Phlomis bruguieri*	Lamiaceae	Aqueous/AP	*n*-hexane–MeOH–acetone (3:6:1)	[39]
76	nepetin	*Santolina chamaecyparissus*	Asteraceae	CH_2Cl_2/AP	MeOH	[65]
77	isoetin	*Taraxacum mongolicum*	Asteraceae	MeOH/AP	MeOH	[21]
78	5,7,4′,5′-tetrahydroxy-2′-methoxyflavone (syn. isoetin 2′-methyl ether)	*Bauhinia galpinii*	Fabaceae	EtOAc/L	acetone–MeOH (1:1)	[66]

79	isoetin-7-*O*-β-D-glucopyranosyl-2′-*O*-α-L-arabinopyranoside		*Taraxacum mongolicum*	Asteraceae	MeOH/AP	MeOH	[21]
80	isoetin-7-*O*-β-D-glucopyranosyl-2′-*O*-α-D-arabinopyranoside		*Taraxacum mongolicum*	Asteraceae	MeOH/AP	MeOH	[21]
81	isoetin-7-*O*-β-D-glucopyranosyl-2′-*O*-α-D-xyloypyranoside		*Taraxacum mongolicum*	Asteraceae	MeOH/AP	MeOH	[21]
82	genkwanin		*Taraxacum mongolicum*	Asteraceae	MeOH/AP	MeOH	[21]
83	genkwanin-4′-*O*-β-D-lutinoside		*Taraxacum mongolicum*	Asteraceae	MeOH/AP	MeOH	[21]
84	albanin A		*Saccharum officinarum*	Poaceae	EtOH (50%)/ST	$CHCl_3$–MeOH (1:1)	[53]
85	australone A		*Saccharum officinarum*	Poaceae	EtOH (50%)/ST	MeOH	[53]
86	5′-geranyl-5,7,2′,4′-tetrahydroxy-flavone		*Saccharum officinarum*	Poaceae	EtOH (50%)/ST	MeOH	[53]
87	chrysoeriol		*Dendrobium ellipsophyllum*	Orchidaceae	MeOH/WP	acetone	[22]
88	4′-methoxyflavone-6-*O*-β-D-glucopyranoside		*Imperata cylindrica*	Poaceae	EtOAc/Rh	MeOH	[27]
89	5-hydroxyflavone		*Imperata cylindrica*	Poaceae	PE/Rh	CH_2Cl_2–MeOH (1:1)	[27]
90	texasin 7-*O*-β-D-glucopyranoside		*Leptadenia pyrotechnica*	Asclepiadaceace	EtOAc/AP	nd	[67]
91	tilianin		*Avena sativa*	Poaceae	EtOH (95%)/bran	MeOH	[64]
92	5-hydroxy-6,7,3′,4′-tetramethoxyflavone		*Citrus aurantium*	Rutaceae	$CHCl_3$/Fl	$CHCl_3$–MeOH (1:1)	[68]
93	formononetin	isoflavone	*Aquilaria sinensis*	Thymelaeaceae	EtOAc/S	MeOH	[57]
94	formononetin-7-*O*-β-D-glucosy1 [1–6] glucoside		*Maackia amurensis*	Fabaceae	EtOAc/B	MeOH–H_2O (6:4)	[69]
95	tectoridin		*Maackia amurensis*	Fabaceae	EtOAc/B	MeOH–H_2O (6:4)	[69]
96	sphaerobioside		*Cudrania tricuspidata*	Moraceae	Aqueous/B	MeOH–H_2O (1:1)	[24]
97	genistein		*Cudrania tricuspidata*	Moraceae	Aqueous/B	MeOH	[24]
98	quercetin	flavonol	*Brachychiton acerifolius*	Malvaceae	EtOH (70%)/L	MeOH–H_2O (1:1)	[33]
			Byrsocarpus coccineus	Connaraceae	*n*-BuOH/L	MeOH	[80]
			Fragaria ananassa	Rosaceae	EtOAc/C	MeOH–H_2O (6:4)	[74]
			Gynura divaricata	Asteraceae	EtOAc/L	$CHCl_3$–MeOH (1:1)	[75]
			Sarcopyramis bodinieri	Melastomataceae	EtOAc/nd	MeOH	[76]
			Cheilanthes tenuifolia	Pteridaceae	MeOH/WP	MeOH (0–60%)–H_2O	[82]
			Albizia amara	Fabaceae	MeOH (70%)/L	MeOH	[83]
			Chionanthus retusus	Oleaceae	EtOAc/Fl	MeOH–H_2O (8:2)	[23]
			Tamarix hohenackeri	Tamaricaceae	EtOAc/AP	MeOH	[77]
			Juniperus chinensis	Cupressaceae	*n*-BuOH/H	$CHCl_3$–MeOH (4:1)	[81]
			Pteris vittata	Pteridaceae	EtOAc/WP	$CHCl_3$–MeOH (1:1)	[78]
			Allium porrum	Amaryllidaceae	MeOH (70%)/AP	MeOH–H_2O (6:4)	[84]

		Athrixia phylicoides	Asteraceae	EtOH/AP	MeOH	[29]
		Taraxacum mongolicum	Asteraceae	MeOH/AP	MeOH	[21]
		Populus davidiana	Salicaceae	EtOAc/W	MeOH–H_2O (3:1, 1:1, 1:3)	[13]
		Paulownia tomentosa	Scrophulariaceae	*n*-BuOH/B	MeOH–H_2O (1:1)	[40]
		Saccharum officinarum	Poaceae	EtOH (50%)/ST	$CHCl_3$–MeOH (1:1)	[53]
		Halimodendron halodendron	Fabaceae	EtOAc/AP	$CHCl_3$–MeOH (1:1)	[79]
		Bauhinia strychnifolia	Fabaceae	Aqueous/S	MeOH	[85]
99	3-*O*-methylquercetin	*Halimodendron halodendron*	Fabaceae	EtOAc/AP	$CHCl_3$–MeOH (1:1)	[79]
100	3,3′-di-*O*-methylquercetin	*Halimodendron halodendron*	Fabaceae	EtOAc/AP	$CHCl_3$–MeOH (1:1)	[79]
101	quercetin-3-*O*-α-rhamnosyl (1→6)-β-D-glucoside (syn. rutin)	*Brachychiton acerifolius*	Malvaceae	EtOH (70%)/L	MeOH–H_2O (1:1)	[33]
		Cheilanthes tenuifolia	Pteridaceae	MeOH/WP	MeOH (0–60%)–H_2O	[82]
		Cinnamomum zeylanicum	Lauraceae	Aqueous/Fr	MeOH–H_2O	[89]
102	quercetin-3-*O*-β-6′′-(*p*-coumaroyl) glucopyranoside-3′-methyl ether (syn. helichrysoside-3′-methyl ether)	*Croton zambesicus*	Euphorbiaceae	EtOH/L	$CHCl_3$ (10–60%)–MeOH	[56]
103	quercetin 3-β-D-glucoside	*Byrsocarpus coccineus*	Connaraceae	*n*-BuOH/L	MeOH	[80]
104	quercetin 3-*O*-α-arabinoside	*Byrsocarpus coccineus*	Connaraceae	EtOAc/L	MeOH	[80]
105	quercetin-3-*O*-β-galactopyranoside	*Bauhinia galpinii*	Fabaceae	EtOAc/L	acetone–MeOH (1:1)	[66]
106	quercetin-3-*O*-α-L-rhamnopyranoside	*Dryopteris filix-mas*	Dryopteridaceae	EtOAc/L	MeOH	[90]
		Cinnamomum zeylanicum	Lauraceae	Aqueous/Fr	MeOH–H_2O	[89]
		Curcuma longa	Zingiberaceae	*n*-BuOH/L	MeOH–H_2O (1:1)	[91]
107	quercetin-3-*O*-β-rhamnoside	*Ficus exasperata*	Moraceae	*n*-BuOH/L	toluene–EtOH (7:3)	[92]
108	quercetin-3-*O*-glucopyranoside	*Indocalamus latifolius*	Poaceae	MeOH/L	MeOH	[42]
		Sambucus ebulus	Adoxaceae	*n*-BuOH/L	MeOH	[93]
109	quercetin-3-*O*-β-D-glucuronide	*Curcuma longa*	Zingiberaceae	*n*-BuOH/L	MeOH–H_2O (8:2)	[91]
		Eugenia jambos	Myrtaceae	EtOH/L	EtOH–H_2O (7:3)	[94]
		Nelumbo nucifera	Nymphaeceae	EtOAc/S	MeOH	[95]
110	quercetin-3-*O*-sambubioside	*Eriobotrya japonica*	Rosaceae	*n*-BuOH/L	MeOH	[96]
111	quercetin 3-*O*-gentiobioside	*Albizia amara*	Fabaceae	MeOH (70%)/L	MeOH–H_2O	[83]
		Oryza sativa	Poaceae	*n*-BuOH/G	MeOH–H_2O (8:2)	[98]
112	quercetin-3-*O*-sophoroside	*Poacynum hendersonii*	Apocynaceae	EtOH (70%)/L	MeOH	[97]
113	quercetin 3-*O*-α-rhamnopyranoside	*Albizia amara*	Fabaceae	MeOH (70%)/L	MeOH–H_2O	[83]
		Allium porrum	Amaryllidaceae	MeOH (70%)/AP	MeOH–H_2O (2:8)	[84]
114	quercetin–3-*O*-α-L-rhap-(1→2)-[α-L-rhap-(1→6)]-β-D-galactopyranoside	*Curcuma longa*	Zingiberaceae	*n*-BuOH/L	MeOH–H_2O (1:1)	[91]
115	quercetin-3-*O*-α-L-rhap-(1→6)-β-D-galactopyranoside	*Curcuma longa*	Zingiberaceae	*n*-BuOH/L	MeOH–H_2O (1:1)	[91]

116	quercetin-3-*O*-α-L-rhap-(1→2)-α-L-rhamnopyranoside	*Curcuma longa*	Zingiberaceae	*n*-BuOH/L	MeOH–H_2O (1:1)	[91]
117	quercetin-3-*O*-β-glucopyranosyl-7-*O*-α-rhamnopyranoside	*Allium porrum*	Amaryllidaceae	MeOH (70%)/AP	MeOH–H_2O (2:8)	[84]
118	quercetin-4′-*O*-β-glucopyranoside	*Allium porrum*	Amaryllidaceae	MeOH (70%)/AP	MeOH–H_2O (4:6)	[84]
119	quercetin-3,7-di-*O*-β-D-di-glucopyranoside	*Taraxacum mongolicum*	Asteraceae	MeOH/AP	MeOH	[21]
120	quercetin-3′,4′,7-trimethyl ether	*Taraxacum mongolicum*	Asteraceae	MeOH/AP	MeOH	[21]
121	quercetin-7-*O*-[β-D-glucopyranosyl(1→6)-β-D-glucopyranoside]	*Taraxacum mongolicum*	Asteraceae	MeOH/AP	MeOH	[21]
122	quercimeritrin (syn. quercetin-7-*O*-glucoside)	*Onopordum alexandrinum*	Asteraceae	EtOAc/Se	MeOH–H_2O (9:1)	[41]
		Cudrania tricuspidata	Moraceae	Aqueous/B	MeOH–H_2O (1:1)	[24]
123	quercetin-7-O-β-D-glucopyranosyl-(2→1)-α-L-rhamnose	*Tridax procumbens*	Asteraceae	EtOAc/WP	nd	[43]
124	dihydroquercetin 7-*O*-β-D-glucoside	*Curcuma longa*	Zingiberaceae	*n*-BuOH/L	MeOH–H_2O (1:1)	[91]
125	quercetrin (syn. quercetin 3-*O*-rhamnoside)	*Camellia japonica*	Theaceae	BuOH/L	$CHCl_3$–MeOH (1:1)	[99]
126	isoquercetin (syn. quercetin 3-β-*O*-glucoside)	*Dorema glabrum*	Apiaceae	EtOAc/AP	MeOH–H_2O (8:2)	[100]
127	quercetin-3-rhamnoside (syn. quercitrin)	*Thuja orientalis*	Cupressaceae	EtOAc/L	MeOH	[58]
		Avena sativa	Poaceae	EtOH (95%)/bran	MeOH	[64]
		Eriobotrya japonica	Rosaceae	*n*-BuOH/L	MeOH	[96]
128	quercetin 3-*O*-β-D-glucopyranoside (syn. isoquercitrin)	*Phyllanthus reticulatus*	Euphorbiaceae	*n*-BuOH/L	MeOH–H_2O (1:1)	[101]
		Poacynum hendersonii	Apocynaceae	EtOH (70%)/L	MeOH	[97]
		Thuja orientalis	Cupressaceae	EtOAc/L	MeOH	[58]
		Juniperus chinensis	Cupressaceae	*n*-BuOH/H	MeOH	[81]
129	isoquercitrin-6-*O*-4-hydroxybenzoate	*Ficus exasperata*	Moraceae	*n*-BuOH/L	toluene–EtOH (7:3)	[92]
130	kaempferol	*Brachychiton acerifolius*	Malvaceae	EtOH (70%)/L	MeOH–H_2O (1:1)	[33]
		Fragaria ananassa	Rosaceae	EtOAc/C	acetone–H_2O (2:1)	[74]
		Gynura divaricata	Asteraceae	EtOAc/L	$CHCl_3$– MeOH (1:1)	[75]
		Gingko biloba	Ginkgoaceae	EtOAc/L	MeOH	[35]
		Albizia amara	Fabaceae	MeOH (70%)/L	MeOH	[83]
		Chionanthus retusus	Oleaceae	EtOAc/Fl	MeOH–H_2O (7:3)	[23]
		Allium porrum	Amaryllidaceae	MeOH (70%)/AP	MeOH–H_2O (8:2)	[84]
		Zygophyllum dumosum	Zygophyllaceae	Aqueous/Sh	MeOH	[110]
		Populus davidiana	Salicaceae	EtOAc/W	MeOH–H_2O (3:1, 1:1, 1:3)	[13]
		Cudrania tricuspidata	Moraceae	Aqueous/B	MeOH	[24]
		Leptadenia pyrotechnica	Asclepiadaceace	EtOAc/AP	nd	[67]
131	7,4′-dimethoxykaempferol	*Tamarix hohenackeri*	Tamaricaceae	EtOAc/AP	MeOH	[77]
132	kaempferol-3-*O*-β-6′′(*p*-coumaroyl)-glucopyranoside (syn. tiliroside)	*Croton zambesicus*	Euphorbiaceae	EtOH/L	$CHCl_3$–MeOH (9:1)	[56]
133	kaempferol 3-*O*-rhamnopyranoside	*Gingko biloba*	Ginkgoaceae	EtOAc/L	MeOH	[35]

134	kaempferol-3-*O*-α-L-rhamnopyranoside	*Curcuma longa*	Zingiberaceae	*n*-BuOH/L	MeOH–H_2O (8:2)	[91]
135	Kaempferol-3-rhamnoside (syn. afzelin, kaempferin)	*Eriobotrya japonica*	Rosaceae	*n*-BuOH/L	MeOH	[96]
		Thuja orientalis	Cupressaceae	EtOAc/L	MeOH	[58]
136	kaempferol-3-rutinoside	*Sideroxylon foetidissimum*	Sapotaceae	PE/L	MeOH	[110]
137	kaempferol 3-*O*-α-arabinoside	*Opuntia dillenii*	Cactaceae	EtOH/Fl	MeOH	[112]
138	kaempferol 3-*O*-α-L-(2-E-p-coumaroyl rhamnopyranoside)	*Platanus acerifolia*	Platanaceae	EtOAc/bud	MeOH	[113]
139	kaempferol 3-*O*-α-L-(2-Z-p-coumaroyl rhamnopyranoside)	*Platanus acerifolia*	Platanaceae	EtOAc/bud	MeOH	[113]
140	kaempferol 3-*O*-α-L-rhamnopyranosyl-(1→6)-β-D-glucopyranoside	*Nelumbo nucifera*	Nymphaeceae	EtOAc/S	MeOH	[95]
141	kaempferol 3-*O*-β-(2″-*O*-α-rhamnosyl)-glucuronide	*Nelumbo nucifera*	Nymphaeceae	EtOAc/S	MeOH	[95]
142	kaempferol 3-*O*-α-L-rhamnopyranosyl-(1→2)-β-D-glucopyranoside	*Nelumbo nucifera*	Nymphaeceae	EtOAc/S	MeOH	[95]
143	kaempferol 3-*O*-β-D-glucuronopyranoside	*Nelumbo nucifera*	Nymphaeceae	EtOAc/S	MeOH	[95]
144	kaempferol 3-*O*-β-D-glucopyranoside (astragalin)	*Allium porrum*	Amaryllidaceae	MeOH (70%)/AP	MeOH–H_2O (6:4)	[84]
		Leptadenia pyrotechnica	Asclepiadaceace	EtOAc/AP	nd	[67]
		Dorema glabrum	Apiaceae	EtOAc/AP	MeOH–H_2O (8:2)	[100]
		Fragaria ananassa	Rosaceae	EtOAc/C	acetone–H_2O (7:3)	[74]
		Avena sativa	Poaceae	EtOH (95%)/bran	MeOH	[64]
145	kaempferol-3-*O*-α-L-rhamnopyranosyl (1‴→6″)-*O*-β-D-glucopyranoside	*Leptadenia pyrotechnica*	Asclepiadaceace	EtOAc/AP	nd	[67]
146	kaempferol-3-*O*-β-D-glucopyranosyl (1‴→6″)-*O*-β-D-glucopyranoside	*Leptadenia pyrotechnica*	Asclepiadaceace	EtOAc/AP	nd	[67]
147	kaempferol 3-*O*-(3''-*E*-*p*-coumaroyl)-α-L-rhamnopyranoside	*Avena sativa*	Poaceae	EtOH (95%)/bran	MeOH	[64]
148	kaempferol 3-*O*-(2''-*O*-*E*-*p*-coumaroyl)-β-D-glucopyranoside	*Avena sativa*	Poaceae	EtOH (95%)/bran	MeOH	[64]
149	8-methoxykaempferol 3-*O*-(6″-malonyl-β-glucopyranoside)	*Crataegus* spp. (Hawthorn)	Rosaceae	MeOH (80%)/L, Fl	MeOH (40-70%)–H_2O	[14]
150	kaempferol 7-*O*-glucoside	*Onopordum alexandrinum*	Asteraceae	EtOAc/Se	MeOH–H_2O (9:1)	[41]
151	kaempferol 7-*O*-β-glucopyranoside	*Allium porrum*	Amaryllidaceae	MeOH (70%)/AP	MeOH–H_2O (6:4)	[84]
152	kaempferol 7-*O*-α-L-rhamnopyranoside	*Avena sativa*	Poaceae	EtOH (95%)/bran	MeOH	[64]
153	isorhamnetin	*Allium porrum*	Amaryllidaceae	MeOH (70%)/AP	MeOH–H_2O (8:2)	[84]
154	isorhamnetin 3-*O*-β-D-rutinoside	*Halimodendron halodendron*	Fabaceae	EtOAc/AP	$CHCl_3$–MeOH (1:1)	[79]
		Opuntia dillenii	Cactaceae	EtOH/Fl	MeOH	[112]
155	isorhamnetin 3-*O*-monoglucoside	*Sambucus ebulus*	Adoxaceae	*n*-BuOH/L	MeOH	[93]
156	isorhamnetin 3-*O*-β-D-glucopyranoside	*Dorema glabrum*	Apiaceae	EtOAc/AP	MeOH–H_2O (8:2)	[100]

157	myricetin (syn. 3,5,7,3′,4′,5′-hexahydroxyflavone)		*Bauhinia galpinii*	Fabaceae	EtOAc/L	acetone–MeOH (1:1)	[66]
158	myricetin 3′,5′-dimethylether 3-*O*-β-D-glucopyranoside		*Nelumbo nucifera*	Nelumbonaceae	EtOAc/stamen	MeOH	[114]
159	myricetin 7-methyl ether 3-*O*-xylopyranosylsyl-(1→2)-α-rhamnopyranoside		*Eugenia jambos*	Myrtaceae	EtOH/L	EtOH–H_2O (3:7)	[94]
160	myricetin-3-*O*-β-galactopyranoside		*Bauhinia galpinii*	Fabaceae	EtOAc/L	acetone–MeOH (1:1)	[66]
161	myricitrin (syn. myricetin 3-*O*-α-rhamnopyranoside)		*Albizia amara*	Fabaceae	MeOH (70%)/L	MeOH–H_2O	[83]
			Thuja orientalis	Cupressaceae	EtOAc/L	MeOH	[58]
162	penduletin		*Plectranthus cylindraceus*	Lamiaceae	MeOH/AP	nd	[115]
163	chrysosplenol D		*Plectranthus cylindraceus*	Lamiaceae	MeOH/AP	nd	[115]
164	sexangularetin		*Fragaria ananassa*	Rosaceae	EtOAc/C	MeOH–H_2O (4:1)	[74]
165	brassicin-4′-*O*-β-D-glucopyranoside		*Oryza sativa* spp. *japonica*	Poaceae	EtOAc/G	acetone (33–100%)–H_2O	[116]
166	5,7,3′-trimethyl-4′-methoxyl-3-O-β-D-flavonoid glucoside		*Tridax procumbens*	Asteraceae	EtOAc/WP	nd	[44]
167	8,3′-dihydroxyl-3,7,4′-trimethoxy-6-O-β-D-flavonoid glucoside		*Tridax procumbens*	Asteraceae	EtOAc/WP	nd	[44]
168	ptevon-3-*D*-glucoside		*Pterocarpus indicus*	Papilionaceae	EtOAc/L	CH_2Cl_2–MeOH (1:1)	[117]
169	sophoflavescenol		*Sophora flavescens*	Fabaceae	CH_2Cl_2/R	CH_2Cl_2–MeOH	[120]
170	leonurusoide E		*Leonurus japonicus*	Lamiaceae	EtOAc/nd	MeOH–H_2O (4:6)	[118]
171	5,4′-dihydroxyflavone-3,6-di-*O*-β-D-glucoside-7-*O*-β-D-glucuronide		*Carthamus tinctorius*	Asteraceae	Aqueous/Fl	H_2O	[121]
172	dillenetin		*Tamarix hohenackeri*	Tamaricaceae	EtOAc/AP	MeOH–H_2O	[77]
173	7-hydroxy-6-methoxyflavone		*Dalbergia cochinchinensis*	Fabaceae	$CHCl_3$/H	CH_2Cl_2–MeOH (1:1)	[10]
174	3-*O*-demethyldigicitrin		*Athrixia phylicoides*	Asteraceae	EtOH/AP	MeOH	[29]
175	tamarixetin 3-O-rhamnopyranoside		*Firmiana simplex*	Malvaceae	EtOAc/SB	MeOH	[119]
176	artemitin		*Taraxacum mongolicum*	Asteraceae	MeOH/AP	MeOH	[21]
177	(3R)-5,7-dihydroxy-8-methyl-3-(2′,4′-dihydroxybenzyl)-chroman-4-one	homoisoflavonoids	*Polygonatum odoratum*	Asparagaceae	EtOH (60%)/Rh	acetonitrile–MeOH (1:1)	[124]
178	(3R)-5,7-dihydroxy-6-methoxy-8-methyl-3-(2′,4′-dihydroxybenzyl)-chroman-4-one		*Polygonatum odoratum*	Asparagaceae	EtOH (60%)/Rh	acetonitrile–MeOH (1:1)	[124]
179	(3R)-5,7-dihydroxy-3-(4′-hydroxybenzyl)-chroman-4-one		*Polygonatum odoratum*	Asparagaceae	EtOH (60%)/Rh	acetonitrile–MeOH (1:1)	[124]
180	(3R)-5,7-dihydroxy-8-methoxy-3-(2′-hydroxy-4′-methoxybenzyl)-chroman-4-one		*Polygonatum odoratum*	Asparagaceae	EtOH (60%)/Rh	acetonitrile–MeOH (1:1)	[124]
181	(3R)-5,7-dihydroxy-8-methyl-3-(4′-hydroxybenzyl)-chroman-4-one		*Polygonatum odoratum*	Asparagaceae	EtOH (60%)/Rh	acetonitrile–MeOH (1:1)	[124]
182	(3R)-5,7-dihydroxy-3-(2′-hydroxy-4′-methoxybenzyl)-chroman-4-one		*Polygonatum odoratum*	Asparagaceae	EtOH (60%)/Rh	acetonitrile–MeOH (1:1)	[124]
183	(3R)-5,7-dihydroxy-6-methyl-3-(4′-hydroxybenzyl)-chroman-4-one		*Polygonatum odoratum*	Asparagaceae	EtOH (60%)/Rh	acetonitrile–MeOH (1:1)	[124]
184	(3R)-5,7-dihydroxy-6-methyl-8-methoxy-3-(4′-hydroxybenzyl)-chroman-4-one		*Polygonatum odoratum*	Asparagaceae	EtOH (60%)/Rh	acetonitrile–MeOH (1:1)	[124]

185	(3R)-5,7-dihydroxy-6,8-dimethyl-3-(4′-hydroxybenzyl)-chroman-4-one		*Polygonatum odoratum*	Asparagaceae	EtOH (60%)/Rh	acetonitrile–MeOH (1:1)	[124]
186	(3R)-5,7-dihydroxy-6-methyl-8-methoxy-3-(4′-methoxybenzyl)-chroman-4-one		*Polygonatum odoratum*	Asparagaceae	EtOH (60%)/Rh	acetonitrile–MeOH (1:1)	[124]
187	cinnamtannin B1 (syn. epicatechin-(2β→O-7,4β→8)-epicatechin-(4β→8) epicatechin)	proanthocyanidin	*Lindera glauca*	Lauraceae	EtOAc/H	MeOH–H_2O (1:1)	[127]
			Cinnamomum zeylanicum	Lauraceae	Aqueous/Fr	MeOH–H_2O	[89]
188	cinnamtannin D1		*Lindera glauca*	Lauraceae	EtOAc/H	MeOH–H_2O (1:1)	[127]
189	procyanidin A1		*Lindera glauca*	Lauraceae	EtOAc/H	MeOH–H_2O (1:1–5:1)	[127]

AP: aerial part; B: bark; Br: bract; C: calix; $CHCl_3$: chloroform; CH_2Cl_2: dichloromethane; EtOAc: ethyl acetate; Fl: flower; Fr: fruit; G: grain; H: heartwood; L: leaf; MeOH: methanol; *n*-ButOH: normal butanol; nd: not determined; P: peel; PE: petroleum ether; R: root; RB: root bark; Rh: rhizome; S: stem; SB: stem bark; Se: seed; Sh: shoot; Sp: sprig; ST: sugarcane top; W: wood; WP: whole plant part; X: xylem
